# Supplementary figures and images for: Obscurin deficiency leads to compensated dilated cardiomyopathy and increased arrhythmias
Source: J Gen Physiol. 2025 May 14;157(4):e202413696. doi: 10.1085/jgp.202413696 (PMC12077377; doi:10.1085/jgp.202413696)

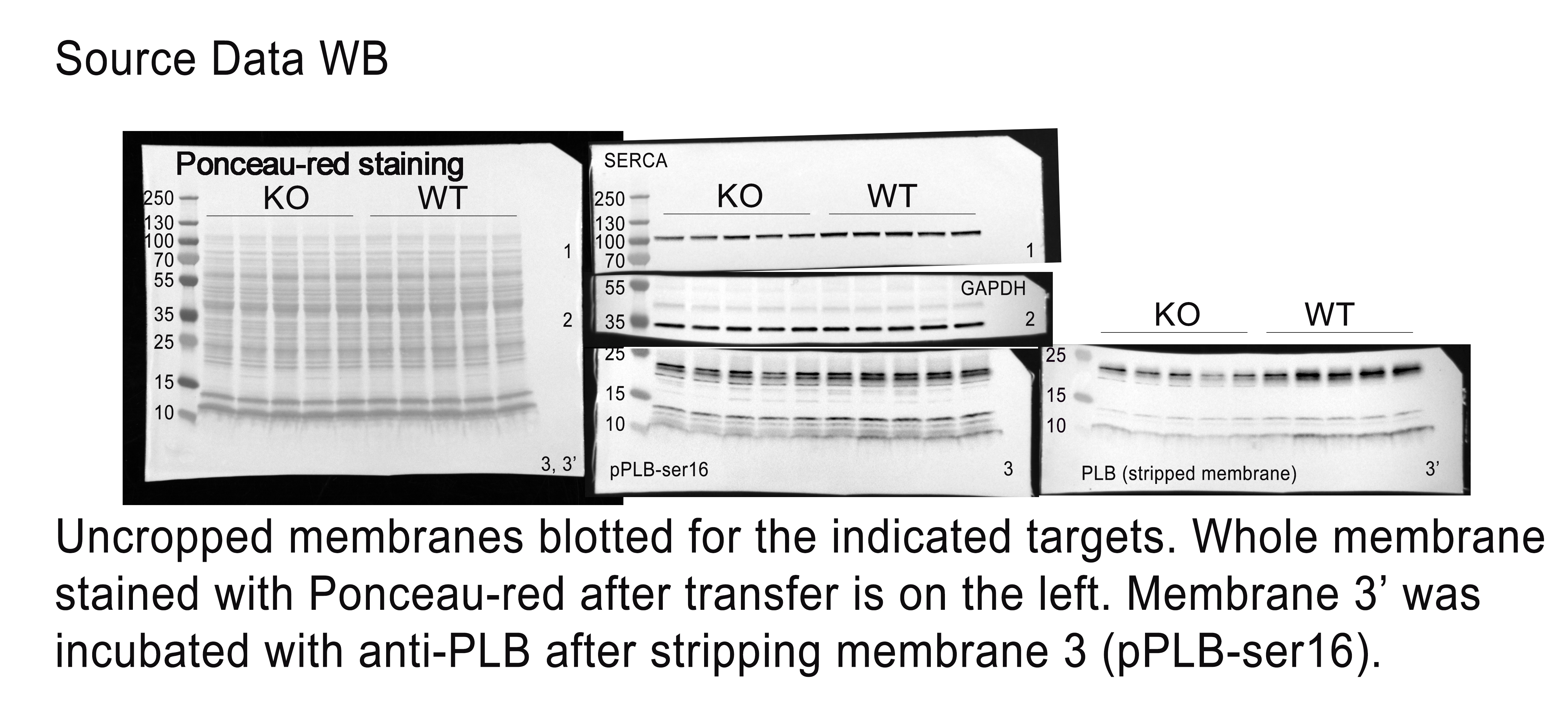

Supplement: SourceData F4 — is the source file for Fig. 4. [file jgp_202413696_sourcedataf4.png]
